# Geographic variation in the treatment of proximal humerus fracture: an update on surgery rates and treatment consensus

**DOI:** 10.1186/s13018-018-1052-2

**Published:** 2019-01-21

**Authors:** Sarah B. Floyd, Joel Campbell, Cole G. Chapman, Charles A. Thigpen, Michael J. Kissenberth, John M. Brooks

**Affiliations:** 1 Center for Effectiveness Research in Orthopaedics, P.O. Box 25571, Greenville, SC 29616 USA; 2 Department of Health Services Policy and Management, University of South Carolina, 915 Greene St., Suite 303C, Columbia, SC 29208 USA; 3 ATI Physical Therapy, 200 Patewood Dr. Suite C250, Greenville, SC 29615 USA; 4 Steadman Hawkins Clinic of the Carolinas, Greenville Health System, 200 Patewood Dr. Suite C100, Greenville, SC 29615 USA

**Keywords:** Surgery, Shoulder, Shoulder fractures, Orthopedics, Medicare, Orthopedic procedures

## Abstract

**Background:**

Using a larger, more comprehensive sample, and inclusion of the reverse shoulder arthroplasty as a primary surgical approach for proximal humerus fracture, we report on geographic variation in the treatment of proximal humerus fracture in 2011 and comment on whether treatment consensus is being reached.

**Methods:**

This was a retrospective cohort study of Medicare patients with an x-ray-confirmed diagnosis of proximal humerus fracture in 2011. Patients receiving reverse shoulder arthroplasty, hemiarthroplasty, or open reduction internal fixation within 60 days of their diagnosis were classified as surgical management patients. Unadjusted observed surgery rates and area treatment ratios adjusted for patient demographic and clinical characteristics were calculated at the hospital referral region level.

**Results:**

Among patients with proximal humerus fracture (*N* = 77,053), 15.4% received surgery and 84.6% received conservative management. Unadjusted surgery rates varied from 1.7 to 33.3% across hospital referral regions. Among patients receiving surgery, 22.3% received hemiarthroplasty, 65.8% received open reduction internal fixation, and 11.8% received reverse shoulder arthroplasty. Patients that were female, were younger, had fewer medical comorbidities, had a lower frailty index, were white, or were not dual-eligible for Medicaid during the month of their index fracture were more likely to receive surgery (*p* < .0001). Geographic variation in the treatment of proximal humerus fracture persisted after adjustment for patient demographic and clinical differences across local areas. Average surgery rates ranged from 9.9 to 21.2% across area treatment ratio quintiles.

**Conclusions:**

Persistent geographic variation in surgery rates for proximal humerus fracture across the USA suggests no treatment consensus has been reached.

## Introduction

Musculoskeletal (MSK) conditions affect around 54% of the US population, account for nearly one in five healthcare visits, and annually exceed $176 billion in direct healthcare costs and $876 billion in indirect costs [1–3]. Yet, remarkably, because of difficulties with randomization and blinding, little randomized controlled trial (RCT) evidence serves as the foundation for this utilization, and there is little consensus on appropriate treatment for many MSK conditions [4–17]. Less than 10% of MSK studies are clinical trials, and of the trials, less than 40% meet minimal guidelines for reporting [9, 10, 18]. This lack of consensus is thought to be the foundation of geographic variation in surgery rates as providers are thought to develop “idiosyncratic clinical rules of thumb” in local areas leading what have been called “surgical signatures” [16, 17]. It is unclear what factors can lead to building treatment consensus for MSK conditions. Here, we theorize that the introduction of new surgical approach for proximal humerus fractures (PHF) will help build consensus. We estimate geographic variation in surgery rates after the introduction of a new surgical procedure for PHF and contrast our results to a study of geographic variation prior to the introduction of the surgical procedure.

## Background

Proximal humerus fractures (PHF) represent 10% of fractures in patients over the age of 65 [19, 20] and can be either non-displaced or displaced in nature. Non-displaced fractures can be successfully treated non-operatively [21], whereas optimal treatment for displaced fractures is more controversial and includes surgical and conservative management approaches. It is unclear which patients stand to benefit the most from surgical treatment [22], and surgery is associated with higher costs [23, 24], surgical and medical complications, and additional revision surgery [25] compared to conservative treatment. Traditional surgical approaches for treating PHF in the elderly included hemiarthroplasty and open reduction and internal fixation (ORIF). However, beginning in 2004 [26–28], with initial approval for use in rotator cuff arthropathy, reverse shoulder arthroplasty (RSA) has been increasingly utilized for treatment of PHF. The RSA procedure offers a more predictable surgical option for elderly patients with complex fractures who may also have underlying glenohumeral joint arthritis and rotator cuff deficiency [28–30]. Previous analysis using data from 2005 demonstrated wide geographic variation in surgical treatment rates [31] and concluded no consensus had been formed as to the right rate of surgery for patients with PHF. However, previous analysis did not include RSA as a surgical approach, excluded watchful waiting patients (those patients with a fracture diagnosis but not receiving formal medical care during the treatment window), had a small sample resulting in suppressed regional estimates, and did not adjust for regional differences in patient characteristics. Therefore, it is not clear how the geographic variation in the management of PHF has changed since the introduction of the RSA procedure, and whether surgeons are closer to reaching a consensus regarding fracture care.

Using a 100% sample of Medicare beneficiaries and comprehensive inclusion criteria, our analysis provides an updated report on the geographic variation in observed surgical treatment for PHF in 2011. Furthermore, a portion of the variation in surgery rates across Hospital Referral Regions (HRRs) that was reported in 2005 could have been the result of differences in underlying populations across HRRs. To assess this, we also estimate adjusted surgery rates across HRRs to account for regional differences in patient demographic and clinical characteristics.

## Methods

### Data and sample

This study used complete Medicare administrative claims data from the years 2010 to 2012 for all Medicare beneficiaries diagnosed with PHF in 2011 (*N* = 130,959). The use of complete Medicare administrative data enabled patient healthcare utilization to be tracked across inpatient and outpatient settings. This project was approved by the University of South Carolina Institutional Review Board.

From this data, individual patients with an x-ray-confirmed diagnosis of PHF in 2011 (ICD-9-CM codes: 812.00, 812.01, 812.02, 812.09, 812.10, 812.11, 812.12, 812.13, 812.19) were identified using Medicare Part B carrier, outpatient and Medpar inpatient claims. Patients with a PHF diagnosis and an x-ray claim within 7 days of the PHF diagnosis date were included in the study. The index date of PHF was defined for each beneficiary as the first date of PHF in 2011. As this study is focusing on treatment for new, acute PHF diagnoses, patients with a PHF diagnosis in the 365 days prior to their index diagnosis in 2011, patients receiving a joint replacement in 365 days prior to their index PHF diagnosis, or patients with a diagnosis of clavicle fracture or hip fracture within 7 days of their index PHF diagnosis were excluded from the study. Additional inclusion criteria applied to assure complete data included (1) continuous enrollment in fee-for-service Medicare Part A and Part B from 365 days prior to 365 days after the index PHF diagnosis and no enrollment in Medicare Part C during the study period, (2) aged 66 years on their surgery date, (3) residence within the continental USA or Hawaii, and (4) complete geographic location information. The minimum age criterion of 66 was used to ensure enrollment in the Medicare system for a year prior to the index surgery.

### Treatment measures

Treatment groups were defined in the 60-day period following the index PHF diagnosis event. Treatment groups were defined as surgical management and conservative management. Patients receiving one of three surgical procedures were classified as surgical management patients. Surgery claims were identified used Part B carrier, outpatient and Medpar Inpatient claims files. The type of surgical procedure patients received was identified using ICD-9-CM procedure and Healthcare Common Procedure Coding System (HCPCS) codes and included RSA (ICD-9-CM codes: 81.88 and HCPCS: 23472), hemiarthroplasty (ICD-9-CM codes: 81.81 and HCPCS: 23470, 23616), or ORIF (ICD-9-CM codes: 79.31 and HCPCS: 23630, 23615, 23670, 23680). Patients with more than one type of surgical procedure indicated on the index surgery date were grouped using a procedure hierarchy based on the complexity of the surgery (RSA > hemiarthroplasty > ORIF). Patients receiving no surgery in the 60-day treatment window were classified as conservative management patients. Complete definitions of treatment variables are provided in the Appendix.

### Patient factors affecting initial treatment choice

Patient demographic characteristics were measured by cross referencing the 2011 Beneficiary Summary Files from Medicare. Specific patient-level variables included age, sex, race, and dual-eligibility status. Concurrent shoulder-related diagnoses made in the 365 days prior to the index PHF in 2011 were used to describe the shoulder health of the fracture population. General patient health was measured using Part A and B Medicare spending in the year prior to the index fracture date, the Charlson Comorbidity Index (CCI), and the Frailty Risk Index (FRI). CCI is a validated measure of burden of disease [32–34]. Comorbidities are weighted from 1 to 6 for mortality risk and disease severity and then summed to form the total CCI score [32–34]. The FRI score is a validated instrument for assessing frailty among older persons [35].

### Analytical approach

To assess the presence of area treatment variation and make comparisons across areas, unadjusted observed surgery rates and risk-adjusted area treatment ratios were calculated at the Hospital Referral Region (HRR) level. HRRs are geographic regions developed by researchers with The Dartmouth Atlas to represent regional healthcare markets for tertiary medical care; each HRR contains at least one major hospital and a minimum population of 120,000. Patients were assigned to an HRR based on residence ZIP code listed in 2011 Medicare Beneficiary Summary data. Unadjusted observed surgery rates were calculated as the proportion of patients in an HRR that received surgery.

Independent relationships between patient-level variables and surgery were estimated by a logistic regression model. The choice to undergo surgery was regressed on patient’s demographic and clinical characteristics. Risk-adjusted area treatment ratios (ATRs) were calculated as the ratio of the number of patients in the HRR who received surgical treatment over the sum across these patients of their predicted probabilities of receiving surgery produced from the logistic regression model [36]. The ATRs are interpreted similar to odds ratios and represent the extent that patients in an HRR were more or less likely to receive a given treatment, independent of their measured characteristics. ATR > 1 for surgery had a local area practice style in which surgery was used at a higher rate than average, given the baseline characteristics of the patients in the HRR. Patients in our full sample were assigned the surgery rate and ATR value based on their residence ZIP code. HRRs were then grouped based on quintiles of surgical ATRs, and average surgery rates were calculated for each group.

Descriptive statistics summarizing patient characteristics across treatment and surgical groups were assessed by the two sample independent *t* test and ANOVA for continuous variables and Pearson’s chi-square for categorical data. The Cochrane-Armitage test was used to assess trends across ATR quintiles. A *p* value of < 0.05 was considered significant. SAS software (version 9.4) was used for data manipulation and statistical analyses; R (version 1.0.153) was used for mapping.

## Results

Table 1 contains the characteristics of our study sample by treatment group. Surgical management was used for 15.4% of the sample, and conservative management was used for 84.6%. Surgical management patients tended to be younger, had fewer comorbidities, a lower frailty index score, and were more likely to be women and white. Additionally, a lower percentage of surgical patients were dual-eligible for Medicaid the month of their index fracture, and a lower percentage had a history of shoulder diagnoses, including shoulder osteoarthritis, rheumatoid arthritis, rotator cuff arthropathy, or avascular necrosis. Surgery patients had lower Medicare spending in the year preceding the index fracture compared to conservative management patients.

**Table 1:** Characteristics of 2011 Medicare proximal humerus fracture patients by treatment group

	Treatment group			*p*
	Total population	Surgical management	Conservative management	
*N*	77,053	11,833	65,220	
Patient demographics				
Male, %	19.9	17.6	20.3	< 0.001
Mean age	80.3	78.1	80.7	< 0.001
Age group, %				< 0.001
66–69	13.0	16.9	12.3	
70–75	20.5	25.5	19.6	
76–79	14.9	17.6	14.4	
80–85	24.8	24.0	25.0	
86+	26.7	16.0	28.6	
Race, %				< 0.001
Asian	0.9	0.7	1.0	
Black	3.1	2.0	3.3	
Hispanic	1.4	1.0	1.4	
Other	1.2	1.1	1.2	
White	93.4	95.1	93.1	
Fully dual eligible^1^, %	13.8	9.3	14.6	< 0.001
Charlson Comorbidity Index^2^, %				< 0.001
0	24.4	28.9	23.6	
1	20.2	21.5	20.0	
2	15.5	15.0	15.6	
3	12.4	12.1	12.4	
4+	27.5	22.5	28.4	
Frailty Risk Index (FRI)				< 0.001
0	34.1	41.4	32.8	
1	25.9	26.8	25.7	
2	15.9	14.7	16.1	
3+	24.1	17.0	25.4	
Shoulder diagnoses in the year prior to index fracture				
Osteoarthritis	25.4	23.7	25.7	< 0.001
Rheumatoid arthritis	8.0	7.4	8.1	0.01
Rotator cuff arthropathy	6.6	6.3	6.6	0.15
Avascular necrosis	0.2	0.3	0.2	0.04
Previous year Medicare spending^3^	$15,623	$12,157	$16,252	< 0.001

Differences across groups assessed by the two sample independent *t* test for continuous variables and Pearson’s chi-square for categorical data

^1^Beneficiary was fully dual-eligible for Medicare and Medicaid during the month of the index fracture

^2^Charlson Comorbidity Index

^3^Total Part A and B payments made by Medicare for the beneficiary over the period of 365 days prior to their index fracture date

Among Medicare patients receiving surgical treatment for their fracture, 22.3% received hemiarthroplasty, 65.8% received ORIF, and 11.8% received RSA. Patients receiving RSA were older and were more likely to have a history of shoulder osteoarthritis, rheumatoid arthritis, or rotator cuff arthropathy. RSA patients had the longest average time from diagnosis to surgery of 11.3 days. More detailed comparisons of surgical groups can be found in Table 2.

**Table 2:** Characteristics of surgically managed Medicare proximal humerus fracture patients by surgical procedure

	Surgical procedure				*p*
	All	Hemi	ORIF	RSA	
*N*	11,833	2644	7792	1397	
Patient demographics					
Male, %	17.6	14.7	18.8	16.7	< 0.001
Mean age	78.1	78.3	77.9	78.8	< 0.001
Age group, %					< 0.001
66–69	16.9	15.0	18.5	11.5	
70–75	25.4	24.4	25.5	27.4	
76–79	17.6	20.2	16.9	17.0	
80–85	24.0	24.5	23.1	27.9	
86+	16.0	15.8	16.1	16.2	
Race, %					0.59
Asian	0.7	0.5	0.8	0.6	
Black	2.0	1.6	2.1	2.4	
Hispanic	1.0	1.1	1.0	1.1	
Other	1.1	1.1	1.2	0.9	
White	95.1	95.7	95.0	94.8	
Fully dual eligible^1^, %	9.3	9.2	9.7	7.2	0.01
Charlson Comorbidity Index^2^, %					0.06
0	28.9	27.6	29.5	28.2	
1	21.5	22.8	21.3	20.1	
2	15.0	15.5	14.7	15.6	
3	21.1	12.5	11.6	14.0	
4+	22.5	21.6	22.9	22.1	
Frailty Risk Index (FRI)					< 0.001
0	41.5	44.4	40.4	42.2	
1	26.8	25.9	26.5	30.1	
2	14.7	14.5	14.8	14.0	
3+	17.0	15.2	18.2	13.7	
Shoulder diagnoses in the year prior to index fracture					
Osteoarthritis	23.7	22.6	23.1	29.3	< 0.001
Rheumatoid arthritis	7.4	6.8	7.4	8.7	0.09
Rotator cuff arthropathy	6.3	6.3	5.2	11.9	< 0.001
Avascular necrosis	0.3	0.4	0.2	0.4	0.08
Previous year Medicare spending^3^	$12,157	$11,269	$12,591	$11,417	0.008
Days to surgery^2^	7.9	8.5	7.0	11.3	< 0.001

Differences across groups assessed by ANOVA for continuous variables and Pearson’s chi-square for categorical data

^1^Beneficiary was fully dual-eligible for Medicare and Medicaid during the month of the index fracture

^2^Days from index diagnosis date to surgery procedure date

^3^Total Part A and B payments made by Medicare for the beneficiary over the period of 365 days prior to their index fracture date

**Fig. 1:**
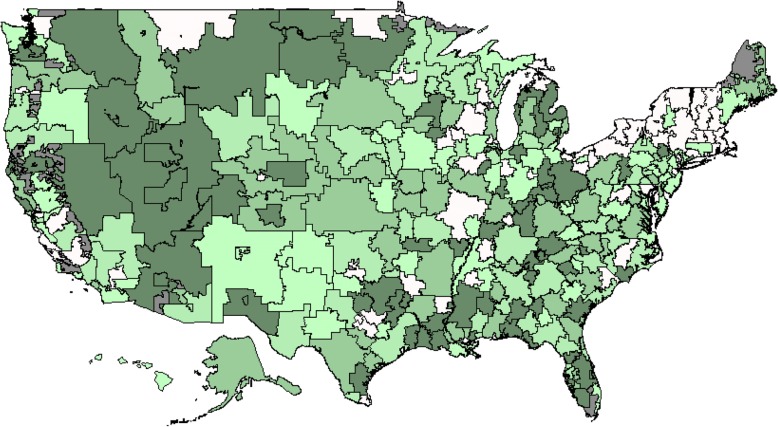
Geographic variation in HRR unadjusted rates of PHF patients treated surgically. Legend:

Figure 1 contains a map of the USA showing unadjusted observed surgery rates in 2011. There was variation observed in the surgical treatment of PHF. The HRR with the highest surgery rate had a surgery rate of 33.3%, whereas the HRR with the lowest surgery rate had a surgery rate of 1.8%. Surgical treatment for PHF appeared to be the highest in the west and upper Midwest regions of the USA.

In the multivariate surgery choice model, males had 0.83 (95% CI 0.79, 0.88) times lower odds of surgery as initial treatment. Being Black and Medicaid dual-eligibility were both associated with lower odds of surgery as initial treatment for PHF. Odds of surgery were 0.64 (0.56, 0.74) times lower, on average, for patients of black race, relative to patients that were white. Patients who were fully dual-eligible for Medicaid had 0.69 times lower odds of surgery (0.65, 0.74). Patients aged 86 years or older had 0.43 (0.40, 0.46) lower odds of receiving surgery relative to patients aged 66–69 years. Patients with a CCI value of 4 or more had 0.84 (0.78, 0.90) times lower odds of surgery compared to having a Charlson Index of 0. A frailty index score of 3 or more was associated with 0.67 (0.62, 0.72) times lower odds of surgery compared to having a frailty index score of 0. Table 3 contains all estimates of relationships between patient-level variables and surgery choice.

**Table 3:** Estimates from logistic models predicting surgical treatment for PHF patients

	Surgery
Male	0.83*** [0.79, 0.88]
Fully dual eligible	0.69*** [0.65, 0.74]
Asian	0.89 [0.70, 1.13]
Black	0.64*** [0.56, 0.74]
Hispanic	0.96 [0.79, 1.16]
Other	0.87 [0.72, 1.05]
Age 70–75	0.95 [0.89, 1.01]
Age 76–79	0.91** [0.85, 0.97]
Age 80–85	0.72*** [0.67, 0.76]
Age 86 plus	0.43*** [0.40, 0.46]
Previous year spending, quintile 2	1.05 [0.98, 1.11]
Previous year spending, quintile 3	1.02 [0.96, 1.09]
Previous year spending, quintile 4	1.12** [1.04, 1.20]
Previous year spending, quintile 5	0.96 [0.88, 1.05]
CCI score 1	0.95 [0.90, 1.01]
CCI score 2	0.89** [0.84, 0.96]
CCI score 3	0.93^+^ [0.87, 1.00]
CCI score 4 or more	0.84*** [0.78, 0.90]
FRI score 1	0.87*** [0.83, 0.92]
FRI score 2	0.83*** [0.78, 0.89]
FRI score 3	0.67*** [0.62, 0.72]
Osteoarthritis	1.06* [1.01, 1.11]
Rheumatoid arthritis	0.95 [0.88, 1.03]
Arthropathy	0.95 [0.88, 1.04]
Avascular necrosis	1.77** [1.18, 2.66]
Observations	77,053

Exponentiated coefficients; 95% confidence intervals in brackets

^+^*p* < .1, *p < .05, **p < .01, ***p < .001

Table 4 shows the distribution of patient characteristics after grouping patients into quintiles of surgical ATRs associated with their HRR of residence. The average percentage of patients who received surgery after PHF varied from 9.9 to 21.2% from lowest to highest ATR quintiles. Few trends were observed in measured baseline factors across local areas.

**Table 4:** Medicare proximal humerus fracture patient characteristics by local area HRR surgical management quintiles

	Quintiles of surgical management area treatment ratios						*p*
	Total population	1	2	3	4	5	
*N*	77,053	15,675	15,648	15,125	15,831	14,774	
Surgical management average area treatment ratio	1	0.65	0.88	0.98	1.13	1.36	
Average surgery rate, %	15.4	9.9	13.7	15.1	17.2	21.1	
Patient demographics							
Male, %	19.9	20.3	19.8	19.8	19.7	19.8	0.27
Mean age	80.3	80.6	80.2	80.1	80.3	80.0	< 0.001
Age group, %							
66–69	13.0	12.1	13.6	13.0	13.3	13.0	
70–75	20.5	19.4	20.4	21.1	20.1	21.7	
76–79	14.9	15.0	14.8	15.0	14.6	15.3	
80–85	24.8	25.4	24.1	25.1	25.0	24.5	
86+	26.7	28.0	27.1	25.7	27.1	25.6	
Race, %							0.04
Asian	0.9	0.8	0.8	0.9	1.6	0.4	
Black	3.1	2.6	3.3	3.1	3.4	3.3	
Hispanic	1.4	1.0	1.1	2.1	1.5	1.0	
Other	1.2	1.2	1.3	1.4	1.4	0.8	
White	93.4	94.4	93.6	92.4	92.1	94.5	
Fully dual eligible^1^, %	13.8	15.5	12.5	14.1	15.4	11.1	< 0.001
Charlson Comorbidity Index^2^, %							
0	24.4	23.9	25.8	23.6	25.2	23.6	0.42
1	20.2	20.1	20.5	20.1	20.5	19.9	0.62
2	15.5	15.2	15.7	15.9	15.3	15.5	0.92
3	12.4	12.5	12.0	12.5	12.0	12.8	0.58
4+	27.5	28.2	26.0	28.0	26.9	28.2	0.45
Frailty Risk Index (FRI)							
0	34.1	33.8	35.2	34.2	34.2	33.2	0.09
1	25.9	26.2	26.0	25.5	25.5	26.3	0.62
2	15.9	16.1	15.5	15.6	15.8	16.3	0.65
3+	24.1	23.8	23.3	24.7	24.5	24.3	0.04
Shoulder diagnoses in the previous 365 days							
Osteoarthritis	25.4	24.3	24.0	26.0	26.0	26.9	< 0.001
Rheumatoid arthritis	8.0	8.4	7.6	8.4	7.6	7.9	0.12
Rotator cuff arthropathy	6.6	6.6	6.3	6.2	6.2	7.5	0.02
Avascular necrosis	0.2	0.2	0.2	0.1	0.2	0.2	0.81
Previous year Medicare spending^3^	$15,623	$16,279	$14,831	$15,738	$15,581	$15,692	< 0.001

Cochrane-Armitage used to asses trends across ATR quintiles

^1^Beneficiary was fully dual-eligible for Medicare and Medicaid during the month of the index fracture

^2^Charlson Comorbidity Index

^3^Total Part A and B payments made by Medicare for the beneficiary over the period of 365 days prior to their index fracture date

**Fig. 2:**
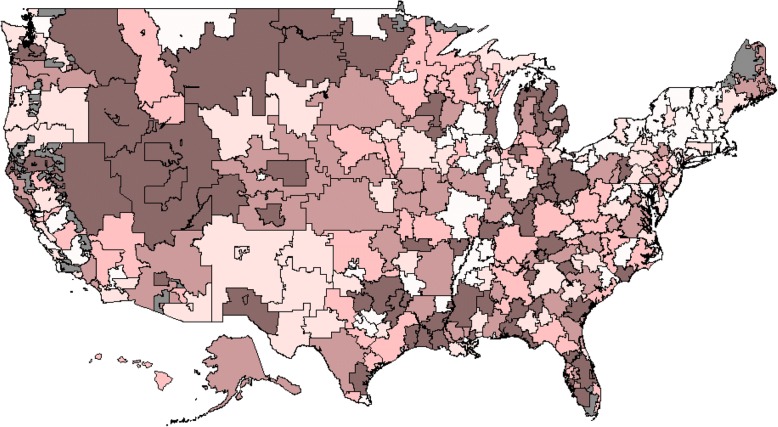
Geographic variation in HRR risk-adjusted surgery area treatment ratios for PHF. Legend:

Figure 2 contains a map of the USA showing the quintile groups of surgical management ATRs. This map shows variation in surgical treatment for PHF at the HRR level. Adjusted estimates of surgery resulted in higher levels of treatment variation than unadjusted rates. Generally, surgical treatment for PHF appeared to be the highest in the Western US and lowest in the Northeast US, although surgery rates varied dramatically within states and regions. Average surgery rates in Fig. 2 were 9.9% in the lighter areas (lowest quintile) and 21.2% in the darker areas (highest quintile).

## Discussion

In this paper, we found evidence that extensive variation in surgery rates existed in 2011 for patients with PHFs and that a treatment consensus had not been reached. The overall surgery rate in our study is consistent to earlier findings [37–39] which suggests that orthopedic surgeons believe there are patients with PHF who will benefit from surgery. The low surgery rate across time also suggests that surgeons recognize that there are detriments associated with surgical treatment and that for many patients the risks associated with surgery (e.g., complications, infections, mortality) may be greater than the expected benefits. Consequently, the relevant question is not whether either surgery or conservative care is “the” effective treatment for all patients with PHF, but rather what is the effective surgery rate of treatments across PHF patients [40–42]. The effective rate can be defined as the surgery rate that when all patients receive their optimal treatments, the treatment that suits them the best [39]. In this paper, we did not find evidence of what the effective surgery rate may be for PHF patients. Since no definitive clinical evidence exists supporting the use of surgery across all PHF patients, our results can help individual surgeons gauge whether their surgery rate for PHF patients are within practice norms.

In our study, 84.6% of Medicare patients with PHFs were treated conservatively. Overall, the frequency at which PHFs were treated surgically remained unchanged from 2005 to 2011 [31]. Bell et al. reported the surgery rate to be 15.7% in 2005. We found the surgery rate to be 15.4% in 2011. Han and colleagues also found the surgery rate remained consistent from 2005 to 2012 [43]. We have no indication that the introduction of the RSA procedure for the treatment of PHF increased surgery rates between 2005 and 2011. This finding is somewhat surprising as one might think the introduction of a new surgical procedure would have expanded surgery as a treatment option to patients previously considered poor candidates for surgical intervention. Alternatively, our results suggest that it is likely that the RSA is becoming the preferred surgical option over the hemiarthroplasty or ORIF procedures for those patients indicated for surgery and that the use of these procedures is on the decline [29]. This finding is corroborated by multiple studies that found the rates of hemiarthroplasty and ORIF utilization were steadily declining from 2009 to 2012 [37, 38, 44].

Bell reported wide ranging geographic variation in the treatment of PHF in 2005 with unadjusted surgical rates varying from 0 to 58% across HRRs [31]. Our study found unadjusted surgery rates ranging from 1.8 to 33.3% across HRRs with surgical treatment being higher in the Western US and lower in the Northeast US. Although our study used a more robust sample than Bell, and we found far less geographic variation compared to that found by Bell in 2005, we still find surgery rates varying dramatically across HRRs in 2011. These findings suggest that a consensus on the effective rate of surgery for PHF patients has not been reached.

The treatment of displaced, three- and four-part fractures in the elderly patient has long been debated and is considered highly controversial. The introduction of a new surgical procedure with favorable outcomes in a difficult-to-treat patient population has potentially reduced some uncertainty surrounding the management of clinically complex patients and increased treatment consensus. Furthermore, the increase in fellowship training for orthopedic surgeons has likely increased the dissemination of information and standardization of practice, further reducing treatment variation [45]. In a study by Acevedo and colleagues, they found that the use of the RSA had risen the fastest among newly trained surgeons and it is likely that training on the RSA device has increased its familiarity and use among younger, fellowship-trained surgeons [29].

This is an observational study where the goal was to assess the presence of treatment variation and assess how the introduction of the RSA-influenced treatment rates for PHF in 2011. One of the major strengths of this study relates to the completeness of the data. Our study sample represents complete data for the entire Medicare population diagnosed with a PHF in 2011.

We recognize that the accuracy of our estimates is contingent on proper diagnosis and procedure coding practices. A weakness of the study is that the use of ICD-9 diagnosis codes does not allow for fracture displacement classification or degree of displacement. It is possible that some of the surgery variation we observed may be related to differences in the proportion of displaced two-, three-, and four-part fractures across HRRs. However, we do not have the reason to suspect that rates of complex fractures occur disproportionately across the country. Based on Table 4, we see that measured patient characteristics were balanced across HRR quintiles. Therefore, we assume that the distributions of unmeasured pertinent clinical characteristics, including fracture complexity, are also consistent across HRRs and the distribution of clinical characteristics would be similar across high and low surgery areas.

## Conclusions

It is not our objective to comment on which rate of surgical treatment is right, but rather document whether variation in surgery rates for patients with PHF remained in 2011. Contrary to our belief, we have no indication that the introduction of the RSA procedure for the treatment of PHF increased surgery rates between 2005 and 2011. And although our study found far less geographic variation compared to that found by Bell in 2005, we still found that surgery rates varied widely across HRRs. In conclusion, geographic variation in the treatment of PHF exists suggesting that a consensus on the effective surgery rate for patients with PHF has not been reached.

## Appendix

Appendix Tables

**Table 5:** Medicare 2011 Proximal Humerus Fracture Sample Inclusion Criteria

Inclusion Criteria	N
Medicare Part B carrier (physician services), outpatient, or medpar (inpatient) claims with a proximal humerus fracture diagnosis from January 1, 2011-December 31, 2011 (ICD-9 Diagnosis codes: 812.00, 812.01, 812.02, 812.09, 812.10, 812.11, 812.12, 812.13, 812.19) (Index diagnosis)	130,959
No Part B carrier, outpatient, or medpar claims with proximal humerus fracture diagnosis in 365-days before the index diagnosis in 2011	107,838
Shoulder x-ray claim (HCPCS codes: 73000, 73010, 73020, 73030, 73050, 73060) in Part B carrier or outpatient revenue center claims within 7 days of index diagnosis (x-ray claim can occur before or after index diagnosis)	95,229
No Part B carrier, outpatient, or medpar claims with a diagnosis of clavicle or hip fracture within 7 days of index diagnosis	86,147
No Part B carrier, outpatient, or medpar claims with total joint replacement procedure in 365-days before the index diagnosis in 2011	85,841
Age 66+ at index diagnosis	84,589
Located within continental United States or Hawaii	84,399
Continuously enrolled in Medicare Parts A and B and never enrolled in HMO, from 365-days prior to index to 365-days after index diagnosis	77,075
Complete HRR data	77,053

**Table 6:** Proximal Humerus Fracture Diagnosis Codes

Diagnosis groups	ICD-9-CM Diagnosis codes
Proximal Humerus Fracture	812.00, 812.01, 812.02, 812.09, 812.10, 812.11, 812.12, 812.13, 812.19

**Table 7:** Hip and Clavicle Fracture Diagnosis Codes

Diagnosis groups	ICD-9-CM Diagnosis codes
Clavicle Fracture	810, 810.0, 810.00, 810.01, 810.02, 810.03, 810.1, 810.10, 810.11, 810.12, 810.13
Hip Fracture	733.14, 733.15, 733.81, 733.82, 808, 808.1, 820, 820.01, 820.02, 820.03, 820.09, 820.10, 820.11, 820.12, 820.13, 820.19, 820.20, 820.21, 820.22, 820.30, 820.31, 820.32, 820.8, 820.9

**Table 8:** X-ray HCPCS Codes

Diagnostic Service	HCPCS codes
Shoulder x-ray	73000, 73010, 73020, 73030, 73050, 73060

**Table 9:** Treatment Groups

Surgery Group	ICD-9-CM Procedure codes	HCPCS Codes
Hemiarthroplasty	81.81	23470, 23616
Reverse Shoulder Arthroplasty	81.88	23472
ORIF	79.31	23615, 23630, 23670, 23680

## Abbreviations

ATRs: Area treatment ratios
CCI: Charlson Comorbidity Index
FRI: Frailty Risk Index
HCPCS: Healthcare Common Procedure Coding System
HRRs: Hospital Referral Regions
MSK: Musculoskeletal
ORIF: Open reduction and internal fixation
PHF: Proximal humerus fractures
RCT: Randomized controlled trial
RSA: Reverse shoulder arthroplasty
